# Associations of serum low-density lipoprotein and systolic blood pressure levels with type 2 diabetic patients with and without peripheral neuropathy: systemic review, meta-analysis and meta-regression analysis of observational studies

**DOI:** 10.1186/s12902-019-0453-5

**Published:** 2019-11-25

**Authors:** Syed Shah Zaman Haider Naqvi, Saber Imani, Hossein Hosseinifard, Qing-Lian Wen, M. Naveed Shahzad, Iqra Ijaz, Youcai Deng, Man Guo, Yong Xu

**Affiliations:** 1 Department of Diabetes and Endocrinology, Affiliated Hospital of Southwest Medical University, Luzhou, Sichuan Province People’s Republic of China; 2 Department of Oncology, Affiliated Hospital of Southwest Medical University, Luzhou, Sichuan Province People’s Republic of China; 3 Research Center for Evidence Based Medicine (RCEBM), Tabriz University of Medical Sciences, Tabriz, Iran; 4 Stem Cell Laboratory, Department of Hematology, Affiliated Hospital of Southwest Medical University, Luzhou, Sichuan Province People’s Republic of China; 5 Sichuan Provincial Center for Gynecological and Breast Diseases, Southwest Medical University, Luzhou, Sichuan Province People’s Republic of China; 6 Institute of Materia Medical, College of Pharmacy, Army Medical University (Third Military Medical University), Chongqing, People’s Republic of China; 7 Luzhou Key Laboratory of Cardiovascular and Metabolic Diseases, Affiliated Hospital of Southwest Medical University, Luzhou, Sichuan Province People’s Republic of China

**Keywords:** Diabetic peripheral neuropathy, Low-density lipoprotein, Systolic blood pressure, Type 2 diabetes, Meta-analysis, Meta-regression

## Abstract

**Background:**

Compositional abnormalities in lipoproteins and cardiovascular risk factors play an important role in the progression of diabetic peripheral neuropathy (DPN). This systematic review aimed to estimate the predicting value of low-density lipoprotein (LDL) and systolic blood pressure (SBP) level in type-2 diabetes mellitus (T2DM) patients with and without peripheral neuropathy. We also tried to determine whether LDL and SBP are associated with an increased collision risk of DPN.

**Methods:**

A systematic search was conducted for eligible publications which explored the LDL and SBP level in T2DM patients with and without peripheral neuropathy. The quality of the included studies was assessed by the QUADAS-2 tool. The standardized mean difference (SMD) with 95% CI of LDL and SBP level were pooled to assess the correlation between LDL and SBP level with DPN. We performed random effects meta-regression analyses to investigate factors associated with an increased collision risk of DPN.

**Results:**

There was a significant association between LDL and SBP with poor prognosis of DPN in those included studies (I_2_ = 88.1% and I_2_ = 84.9%, respectively, Both *P < 0.001*). European T2DM patients have higher serum level of LDL in compare with the European DPN patients (SMD = 0.16, 95% CI: − 0.06 - 0.38; *P <* 0.001). SBP level was associated with a 2.6-fold decrease in non-DPN patients of T2DM (SMD = − 2.63, 95% CI: − 4.00 - -1.27, *P <* 0.001). Old age European T2DM patients have significantly high risk for diabetes drivers. Furthermore, the results of the case-control study design model are more precise to show the accuracy of SBP in Asian T2DM patients.

**Conclusion:**

Our finding supports the LDL and SBP status could be associated with increased risk of peripheral neuropathy in T2DM patients.

## Background

Diabetic peripheral neuropathy (DPN) is the major debilitating chronic complication of type-2 diabetes mellitus (T2DM), with an estimated lifetime incidence of about 47% of patients with T2DM and 21% of pre-diabetes [1, 2]. Over the last 10 years, the overall annualized incidence rates of DPN have been rapidly increased from 9.4 to 61.8% in a population of diabetic neuropathies. The typical DPN is a distal symmetric polyneuropathy that is characterized by the neuropathy experience symptoms such as hyperalgesia, paresthesia, amputation, burning pain, stabbing sensations, hyperesthesia, and deep aching pain [3].

Many studies have confirmed that DPN progression is strongly associated with cardiovascular and metabolic risk factors, such as obesity, hypertension, hyperfibrinogenemia, hypercholesterolemia and dyslipidemia [4–7]. Also, compositional abnormalities in lipoproteins play an important role in the progression of atherosclerosis in T2DM with nephropathy. Accumulated evidence suggests that improvement in glycemic control and blood pressure control have all helped to reduce the incidence and progression of diabetic neuropathy [8–12]. T2DM patients suffering from peripheral neuropathy have different low-density lipoprotein (LDL) and systolic blood pressure (SBP) profiles that potentially influence the occurrence of DPN [13]. It has been widely reported that different serum level of LDL is related to poor survival and prognosis of polyneuropathy in diabetic patients [14]. Clinically, the difference values of LDL and SBP can be used for early diagnosis and differentiation of T2DM patients with and without peripheral neuropathy [15–17]. Although, intervention treatment strategies have thus far not revealed that a specific pharmacologic approach can prevent T2DM neuropathy.

Many time series meta-regression analysis tried to determine whether LDL and SBP are associated with an increased collision risk of DPN [18–21]. It is interesting to note that collision risk in DPN drivers changed over time in old age of T2DM patients [22]. Certainly, understanding the role of LDL and SBP in pathogenesis and collision risk of diabetic neuropathy could help to develop effective treatments and road safety regulations for type 2 diabetic neuropathy. Despite numerous experimental studies, the prognostic value of LDL and SBP for survival in T2DM patients with DPN is still controversial and inconclusive [21].

Hence, we conducted a quantitative systematic review along with a comprehensive meta-analysis investigation among a large sample of T2DM patients to estimate the predicting value and prognostics accuracy of LDL and SBP in T2DM patients with and without peripheral neuropathy. Furthermore, we planned to assess the association between LDL and SBP deficiency and DPN in T2DM. Secondary objectives of this study were to see whether LDL and SBP are associated with an increased collision risk of DPN. We tried to test the effect of age and years of disease on the overall collision risk for diabetes drivers.

## Methods

### Search strategy and study selection

A comprehensive systematic search from the literature published in English was performed by querying the MEDLINE electronic database including PubMed, Embase, Wiley Online Library, ISI Web of Science, Cochrane library and VIP-Google Scholar to identify all the relevant studies. Electronic medical literature databases searched and retrieved prior to Feb 20, 2019, by three researchers separately (SSN, MG, and MNS). Definitely, main terms were linked using Boolean “AND” to identify all the relevant reports and different spelling and synonyms were combined using Boolean “OR”. The search string was conducted without regional restrictions by using MeSH terms and following main headline terms or free word based on the research question (both the UK and US spellings), such as: “diabetic peripheral neuropathy OR type 2 diabetes” AND “low density lipoprotein OR systolic blood pressure” AND “prognosis OR survival OR outcome”.

### Inclusion/exclusion criteria

The current meta-analysis included all prospective and human randomized controlled trials studies that were considered eligible if they met the following criteria: (*i*) Observational comparative studies relating to diabetic peripheral neuropathy in type 2 diabetes; (*ii*). Patients diagnosed with confirmed type 2 diabetes according to American Diabetes Association (ADA) or World Health Organization (WHO) criteria; (*iii*) Studies which provide LDL level for T2DM with and without peripheral neuropathy; (*iv*) Studies which provide SBP level in both T2DM patients with and without peripheral neuropathy. Likewise, we excluded all non-comparative, review, conference abstracts, meeting, comments, and family-based studies, and unrelated articles, in vitro and animal studies. In addition, we excluded studies only mentioning “diabetes” (with no discrimination of subtypes), studies on prediabetes population (less than 18 years of age), duplicate studies, continued work of previous publications, and poor quality studies, as well as those with incomplete and/or missing data.

### Data extraction

All selected articles were reviewed independently by two investigators (QW and II) according to PICO principle [23] and any inconsistencies or disagreements in a search process were resolved through consultations and debate. If they could not reach an acceptable consensus, a third partner (SI) would resolve these disagreements according to the original data. The following demographics and clinicopathological key components of all qualified studies were recorded: first author’s name, publication year, country origin, ethnicity, total cases, study design, the total number of T2DM patients with and without peripheral neuropathy, baseline levels of LDL and SBP. Moreover, we e-mailed the corresponding authors of the selected articles to obtain any missing or additional information and copies of the original data required for the meta-analysis. If the above data were not cited in the original study or no replay was received by email, the item was reported as “not reported (NR)”.

### Quality assessment

This present systematic review and meta-analysis were performed in accordance with the guidelines of Preferred Reporting Items for Systematic Reviews and Meta-analysis (PRISMA) [24, 25]. All eligible studies were assessed according to the Newcastle-Ottawa scale (NOS) [26] and Agency for Healthcare Research and Quality (AHRQ) criterion [27]. Diagnostic accuracy of studies was assessed by Quality Assessment of Diagnostic Accuracy Studies 2 (QUADAS-2) protocols, tool in patient selection, index test, reference standard, and flow timing [28, 29]. QUADAS-2 was assessed to determine the quality of all the studies by three authors (SSN, MG, and MNS) and any disagreements were resolved through a discussion. Also, the risk of bias was calculated according to the criteria from the Cochrane Collaboration’s tool (Cochrane handbook for systematic reviews of interventions version 5.1.0.). Briefly, on Cochrane Collaboration’s tool, each of the assessment has seven questions with the answered with “yes”, “no”, or “unclear”. The answer of “yes” means that a study’s risk bias can be judged as low, while “no” and “unclear” mean that the risk of bias can be referred to as high. The quality assessment table for each selected study is sorted in Table S1 (Additional file 1: Table S1),

### Statistical analysis

The current systematic meta-analysis was performed using Comprehensive Meta-Analysis software (the USA, version 2.2.064). Data were presented as mean ± Std. deviation (SD) or median (range), as well as a description of qualitative variables such as number and percentage. We calculated the standardized mean difference (SMD) with 95% confidence intervals (CIs) to evaluate the difference between LDL and SBP level between DPN and non-DPN in patients with T2DM. The chi-square-based Q-test was applied to testify between-study heterogeneity. They were considered statistically heterogeneous if they displayed *P <* 0.05 and/or *I*^*2*^ *>* 50% [30]. Subgroup analysis was conducted to determine the source of existing heterogeneity between the serum LDL and SBP markers and available sub analysis such as race and study design. Meta-regression was weighted by a number of subjects unless otherwise specified. Random effects meta-regression using serum levels data for LDL and SBP, participant age (centered on mean) and year in which the study was published considering the first study included in the analysis as being published in the year 2004. Publication bias was evaluated by Begg’s funnel plots [31] and Egger’s regression test [32]. A value of “Pr > |z|” less than 0.05 was considered to be potential publication bias. All reported *P* values were two-sided and *P*-values < 0.05 was considered statistically significant.

## Results

### Literature search

**Fig. 1:**
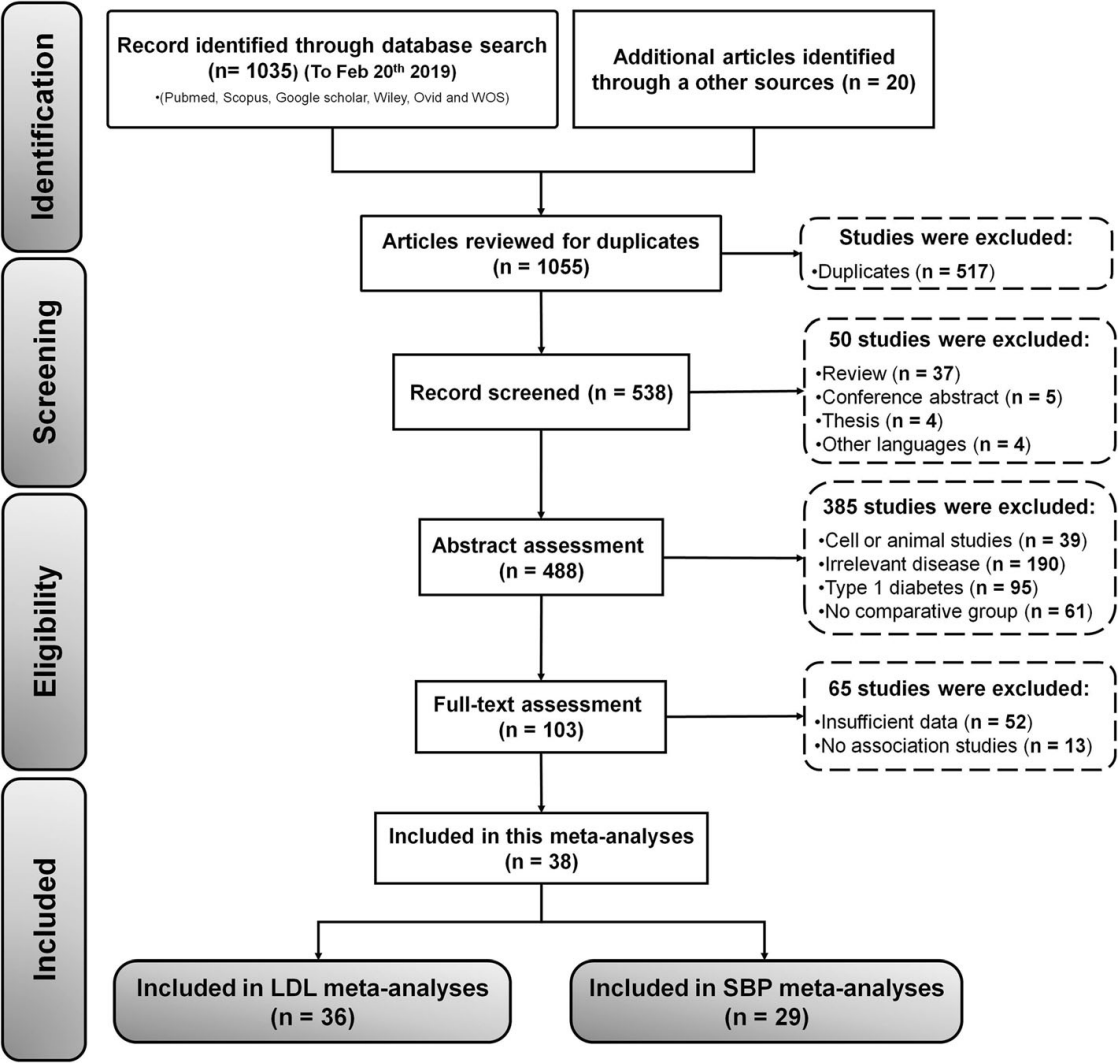
PRISMA diagram for selection of studies (n = number of studies)

The detailed flowchart of the screening and selection process in the PRISMA flow diagram is shown in Fig. 1. Afterward 1055 potentially relevant studies exclusion, 1055 of papers eligible for inclusions were confirmed with the initial search strategy. After initial screening, 517 studies were removed due to duplication studies, Of the 538 candidate studies, 46 studies were excluded due to unrelated study design, and 488 articles were left for abstract assessment. After carefully reviewing titles and abstracts, 385 studies were precluded for obvious irrelevance disease, cell or animal studies data, lack of comparative group. Then, 103 studies were chosen for full-text valuation. Of the remaining 103 full-text candidate articles, 65 potential studies were excluded, due to insufficient and useable data. Finally, 38 studies were selected to find a relationship between LDL and SBP levels and risk of diabetic peripheral neuropathy [4–17, 22, 33–55]. Of the 38 finalized studies, 2 studies [22, 55] and 7 studies [6, 10, 14–16, 35, 47, 50, 52] were excluded involving insufficient data to find a relationship between LDL and SBP levels and risk of DPN, respectively. Hence, in this current meta-analysis, only 29 articles were attempts to find a relationship between SBP levels and DPN in type 2 diabetes mellitus patients.

### Quality assessment

To evaluate the methodological quality of selected studies, we applied the NOS and AHRQ criterion. The detailed quality assessment of eligible studies according to design, enrollment scheme for participants, the credibility of results, assessment of confounding factors and made their individual reports, were summarized individually in Table S1 (Additional file 1: Table S1). Overall, all 38 selected studies in the current meta-analysis were judged to be at moderate to high risk of bias, with scores ≥7 points. The average NOS score was 7.68 out of 10, that was pretty categorized in the high-quality evaluation standards of the Cochrane Reviewers’ Handbook. Furthermore, QUADAS-2 results confirmed that significant bias was not presented in the current meta-analyses (Additional file 2: Figure S1). The reviewers’ decisions about each risk of bias and applicability concerns graph presented as percentages across selected studies. More than half of the included studies were rated as low risk for most parameters of the bias risk (49.83%) and applicability concerns (60.54%). In this study, no significant bias and applicability concerns were found in all selected studies (Additional file 2: Figure S1).

### Study and patient’s characteristics

Characteristics of all relevant studies included in this systematic review and meta-analysis are summarized in Table 1. A total of 355,438 patients were included in these studies, and the median trial sample size was 161,734 patients, between 2004 and 2018. Gender subgroups among 354,088 patients, 168,095 (47.4%) and 185,993 (52.6%) patients were male and female, respectively. Most studies used a cross-sectional study (63.10%) deign for measuring LDL and SBP, respectively. The mean age of the participants was 60.11 ± 10.00 years. The mean duration of diabetes was 6.50 ± 2.80 years; and the mean level of LDL and SBP were 2.82 ± 0.80 mmol/L and 134.81 ± 15.10 mmHg, respectively (Table 1).

**Table 1:** Demographics information of included studies

First author (Ref.)	Year	SS	Gender (M/F)	Mean Duration of DM (Months)	Population (ethnicity)	Study design	NOS^a^
Zhao W. et al. [4]	2016	469	283/186	120	China (A)	Cross-sectional	8
Wu F. et al. [5]	2017	1134	645/489	78	China (A)	Cross-sectional	8
ALMA R. et al. [6]	2014	82	52/30	99.6	Canada (AM)	Case control	8
Yang J. et al. [7]	2017	1511	743/768	105	China/A	Cross-sectional	8
Bilir B. et al. [8]	2016	99	47/52	NA	Turkey/A	Clinical study	7
Sharon L. et al. [9]	2017	1981	1001/980	144	Singapore/A	Cross-sectional	8
Yong J. et al. [10]	2015	180	96/84	135.6	China/A	Case control	8
Su J. et al. [11]	2018	563	299/264	67.2	China/A	Cross-sectional	8
Zhang Y. et al. [12]	2017	1059	589/470	102.7	China/A	Case control	7
Won J. et al. [13]	2012	3999	1939/2060	121.2	Korea/A	Cross-sectional	7
Lin X. et al. [53]	2017	200	123/77	109	China/A	Cross-sectional	8
Qiao X. et al. [54]	2017	185	79/106	123	China/A	Cross-sectional	8
Sadosky A. et al. [34]	2014	323,378	151,927/171451	NA	USA/NA	Retrospective	8
YM. S. et al. [55]	2018	982	497/485	64	China/A	Cross-sectional	7
Khawaja N. et al. [56]	2018	1003	480/523	120	Jordan/A	Cross-sectional	7
Zhang Q. et al. [57]	2018	254	127/127	138	China/A	Case control	8
Jangh M. et al. [58]	2006	810	289/521	98.4	Iran/A	Cross-sectional	8
Kim S. et al. [59]	2013	1338	593/745	145	Korea/A	Cross-sectional	8
Bansal D. et al. [14]	2014	2006	989/1017	104.4	India/A	Cross-sectional	8
Buraczy M. et al. [15]	2016	1244	594/650	153.6	Poland/EU	Case control	8
Luo Y. et al. [16]	2015	412	233/179	150	China/A	Cross-sectional	7
Ren Z. et al. [17]	2015	787	395/392	179.4	China/A	Case control	8
Andersen S. et al. [44]	2018	1256	735/521	156	Denmark/EU	Cohort study	7
Anastasi T. et al. [45]	2017	381	220/161	147	Greece/EU	Cross-sectional	8
Xu T. et al. [46]	2017	537	161/376	NA	China/A	Observational	8
Zhu T. et al. [52]	2014	64	31/33	NA	China/A	Cross-sectional	8
Deng W. et al. [51]	2014	202	115/87	98	China/A	Cross-sectional	8
Thainá R. et al. [50]	2018	426	162/264	92	Brazil/SA	Cohort study	7
Miric D. et al. [35]	2016	80	33/47	72	Serbia/EU	Case control	7
Hussain G. et al. [42]	2013	86	38/48	96	India/A	Cross-sectional	7
Li L. et al. [43]	2014	3359	1607/1268	91	China/A	Cross-sectional	8
Pai Y. et al. [36]	2018	2837	1661/1186	130.4	Taiwan/A	Cross-sectional	7
Xu F. et al. [48]	2014	90	46/44	66	China/A	Cross-sectional	8
Wang H. et al. [37]	2013	207	88/119	NA	China/A	Case control	7
Wang H. et al. [40]	2012	261	124/137	76.9	China/A	Case control	8
Pai Y. et al. [39]	2018	626	333/293	184.8	Taiwan/A	Case control	8
Mao F. et al. [22]	2018	950	555/395	106.2	China/A	Cross-sectional	8
Hoque S. et al. [33]	2016	400	166/234	72	Bangladesh/A	Cross-sectional	8

Abbreviations: *Ref.* reference, *SS* sample size, *M* male, *F* femal, *AM* amaricen, *A* Asian, *EU* europea, *NOS* Newcastle-Ottawa scale, *NA* not avalibale

^a^The quality of non-randomized studies will be appraised using the Newcastle-Ottawa scale (NOS), categorized into three groups: the selection of the study groups; the comparability of the groups; as well as the ascertainment of either the exposure or outcome of interest for case-control or cohort studies respectively

The participants were divided into 2 groups: that T2DM without neuropathy (*n* = 309,197) and patients with DPN without pain (*n* = 44,891), representing an overall DPN prevalence of 12.67% (Table 2). According to Table 2, a total of 36 studies were included in the analysis of LDL comprising of 354,088 patients (309,197 cases without and 44,891 cases with DPN). Subgroup analyses for LDL were based on the continent were done by diving studies done from Asia (*n* = 29, 80.6%), Europe (n = 4, 11.2%), and America (n = 3, 8.4%). Based on study type, there were three subgroups: cross-sectional studies (*n* = 22, 61.2%), case-control studies (*n* = 11, 30.6%) and cohort studies (n = 3, 8.4%). Analysis for SBP comprised of 22 studies. For SBP, a total number of subjects, in the final analysis, was 25,022 cases including 16,969 cases without and cases 8053 with DPN. For the sake of subgroup analyses of SBP, most of the studies were conducted in people of the Asian race, tracked by 26 studies (72.2), 2 studies (5.6%) in European countries, one study in the USA (2.8%). In our study, there were no data from African populations. Of all the studies, 21 cross-sectional studies (58.4%), 6 case-control studies (16.67%) and 2 cohort studies (5.56%) were attempts to find a relationship between SBP and risk of type 2 diabetic peripheral neuropathy. The analysis of these 36 studies was performed consistently without any studies from African populations.

**Table 2:** Main clinicopathological characteristics of all relevant studies

First author (Ref.)	T2DM without DPN					T2DM with DPN				
	N (M/F)	Age (yrs.)	LDL (mmol/L)	SBP (mmHg)	*P*-value*	N (M/F)	Age (yrs.)	LDL (mmol/L)	SBP (mmHg)	*P*-value**
Zhao W. et al. [4]	365 (230/135)	59.48	2.75 ± 0.86	130.29 ± 15.93	0.01	104 (53/51)	66.94	2.62 ± 0.82	135.81 ± 17.65	0.133
Wu F. et al. [5]	560 (338/222)	53.20	2.84 ± 0.96	129.10 ± 17.30	<0.01	574 (307/267)	61.00	2.66 ± 0.95	134.4 ± 18.40	0.002
ALMA R. et al. [6]	12 (6/6)	53.60	2.20 ± 0.70	NA	NA	70 (46/24)	60.23	2.30 ± 1.25	NA	NA
Yang J. et al. [7]	1287 (626/661)	58.61	2.78 ± 1.01	132.06 ± 20.54	0.03	224 (117/107)	66.58	2.75 ± 0.99	134.72 ± 22.25	0.438
Bilir B. et al. [8]	53 (24/29)	58.85	3.59 ± 1.40	128.97 ± 11.73	NA	46 (23/23)	56.91	3.15 ± 0.99	131.62 ± 14.25	NA
Sharon L. et al. [9]	1767 (866/901)	57.20	2.76 ± 0.83	137.60 ± 18.70	< 0.01	214 (135/79)	60.10	2.71 ± 0.81	146.00 ± 20.60	0.45
Yong J. et al. [10]	90 (50/40)	54.90	2.90 ± 0.88	NA	NA	90 (46/44)	54.10	2.73 ± 0.82	NA	0.18
Su J. et al. [11]	461 (248/213)	56.00	2.67 ± 0.82	134.00 ± 17.00	0.10	102 (51/51)	58.40	2.60 ± 0.86	137.00 ± 19.00	0.44
Zhang Y. et al. [12]	642 (342/300)	60.03	2.92 ± 0.96	134.29 ± 19.14	0.00	417 (247/170)	61.66	2.95 ± 1.15	142.50 ± 22.39	0.60
Won J. et al. [13]	2661 (1346/1315)	59.40	2.60 ± 0.09	127.00 ± 14.40	0.39	1338 (593/745)	62.70	2.40 ± 0.90	127.50 ± 15.50	0.02
Lin X. et al. [53]	123 (NA)	52.83	3.19 ± 0.94	135.52 ± 17.84	< 0.05	77 (NA)	60.79	3.39 ± 1.28	143.12 ± 22.72	> 0.05
Qiao X. et al. [54]	128 (53/75)	62.30	2.96 ± 0.77	133.00 ± 16.00	0.03	57 (26/31)	64.50	2.98 ± 1.09	141.00 ± 13.00	0.90
Sadosky A. et al. [34]	288,328 (134,761/153580)	61.40	2.62 ± 0.85	NA	NA	35,050 (17,166/17871)	64.80	2.37 ± 0.84	NA	0.00
YM. S. et al. [55]	785 (403/382)	54.60	2.52 ± 0.81	132.00 ± 15.00	0.07	197 (94/103)	57.10	2.5 ± 0.88	135.00 ± 17.00	0.76
Khawaja N. et al. [56]	607 (201/195)	62.70	2.61 ± 0.78	142.40 ± 18.10	<0.01	396 (279/328)	57.80	2.64 ± 0.80	138.30 ± 17.50	<0.01
Zhang Q. et al. [57]	159 (79/80)	59.75	2.68 ± 0.95	133.30 ± 18.14	0.03	95 (48/47)	64.02	2.54 ± 1.16	138.85 ± 21.18	0.29
Jangh M. et al. [58]	202 (NA)	49.40	3.39 ± 1.03	125.00 ± 21.30	<0.05	608 (NA)	53.80	3.37 ± 0.95	129.20 ± 23.40	0.90
Kim S. et al. [59]	761 (360/401)	62.50	2.44 ± 0.84	128.00 ± 16.00	0.41	577 (233/344)	63.60	2.45 ± 0.88	127.00 ± 15.00	0.98
Bansal D. et al. [14]	1420 (701/719)	52.50	2.79 ± 1.03	NA	NA	586 (288/298)	57.10	2.379 ± 1.20	NA	<0.01
Buraczy M. et al. [15]	838 (400/438)	62.90	2.80 ± 1.09	NA	NA	406 (194/212)	63.00	2.80 ± 1.05	NA	1.00
Luo Y. et al. [16]	178 (107/71)	53.00	2.70 ± 0.90	NA	NA	234 (126/108)	59.00	2.60 ± 0.90	NA	0.43
Ren Z. et al. [17]	385 (191/194)	66.40	2.50 ± 0.80	133.00 ± 18.00	0.46	402 (204/198)	63.80	2.60 ± 0.80	132.00 ± 18.00	0.15
Andersen S. et al. [44]	1178 (693/485)	60.90	3.42 ± 0.12	148.00 ± 21.00	<0.05	78 (42/36)	60.60	3.10	150.50 ± 14.23	<0.05
Anastasi T. et al. [45]	274 (143/131)	63.30	3.34 ± 1.40	141.20 ± 19.70	<0.01	107 (77/30)	66.10	3.00 ± 1.10	150.40 ± 22.90	0.02
Xu T. et al. [46]	397 (111/286)	62.34	3.07 ± 0.90	137.14 ± 19.72	0.27	160 (50/90)	62.38	3.13 ± 1.07	135.04 ± 21.36	0.50
Zhu T. et al. [52]	32 (16/16)	54.38	2.64 ± 0.76	126.96 ± 11.30	NA	32 (15/17)	56.00	2.94 ± 1.23	128.97 ± 11.97	NA
Deng W. et al. [51]	80 (43/37)	60.68	2.71 ± 0.63	127.86 ± 16.36	0.08	122 (72/49)	60.69	2.76 ± 0.70	132.64 ± 20.26	0.92
Thainá R. et al. [50]	258 (101/157)	66.00	2.94 ± 1.01	142.00 ± 22.00	0.07	168 (61/107)	70.00	3.04 ± 0.99	146.00 ± 23.00	0.36
Miric D. et al. [35]	51 (20/31)	61.50	3.88 ± 1.32	NA	NA	29 (13/16)	62.90	4.01 ± 1.45	NA	NA
Hussain G. et al. [42]	22 (10/12)	51.90	2.29 ± 0.34	132.82 ± 10.63	NA	64 (28/36)	54.68	2.50 ± 0.59	140.00 ± 39.14	<0.05
Li L. et al. [43]	2246 (1148/1098)	57.00	3.10 ± 1.19	135.70 ± 18.85	<0.01	1113 (459/654)	62.30	3.09 ± 1.12	139.27 ± 19.15	0.82
Pai Y. et al. [36]	2223 (1280/943)	61.90	2.64 ± 0.81	NA	NA	624 (381/243)	71.40	2.51 ± 0.80	NA	0.02
Xu F. et al. [48]	45 (21/24)	58.70	2.70 ± 0.80	139.00 ± 18.00	0.19	45 (25/20)	59.80	2.30 ± 0.60	134.00 ± 18.00	0.01
Wang H. et al. [37]	78 (34/44)	60.59	3.03 ± 0.68	NA	NA	129 (54/75)	61.53	2.96 ± 0.88	NA	NA
Wang H. et al. [40]	150 (73/77)	60.80	3.60 ± 2.20	135.00 ± 19.70	NA	101 (51/50)	61.80	3.00 ± 1.90	127.00 ± 16.50	NA
Pai Y. et al. [39]	351 (186/165)	72.50	2.54 ± 0.72	133.20 ± 15.00	0.15	275 (147/128)	73.50	2.49 ± 0.73	131.25 ± 14.90	0.93
Mao F. et al. [22]	686 (398/288)	57.73	NA	127.81 ± 13.10	0	264 (157/107)	63.78	NA	131.82 ± 15.33	NA
Hoque S. et al. [33]	304 (NA)	49.68	NA	127.12 ± 12.62	0.20	96 (NA)	51.93	NA	129.33 ± 14.66	NA

*P-values is calculated LDL mean between the T2DM with and without DPN

**P-values is calculated SBP mean between the T2DM with and without DPN

Abbreviations: N, number; M, male; F, femal; T2DM, type-2 diabetes mellitus; DPN, diabetic peripheral neuropathy; LDL, low-density lipoprotein; SBP, systolic blood pressure; mmol/L, millimole per liter; mmHg, millimeter of Mercury; NA, not avalibale

## Meta-analysis results

### Association of serum LDL level with DPN

**Fig. 2:**
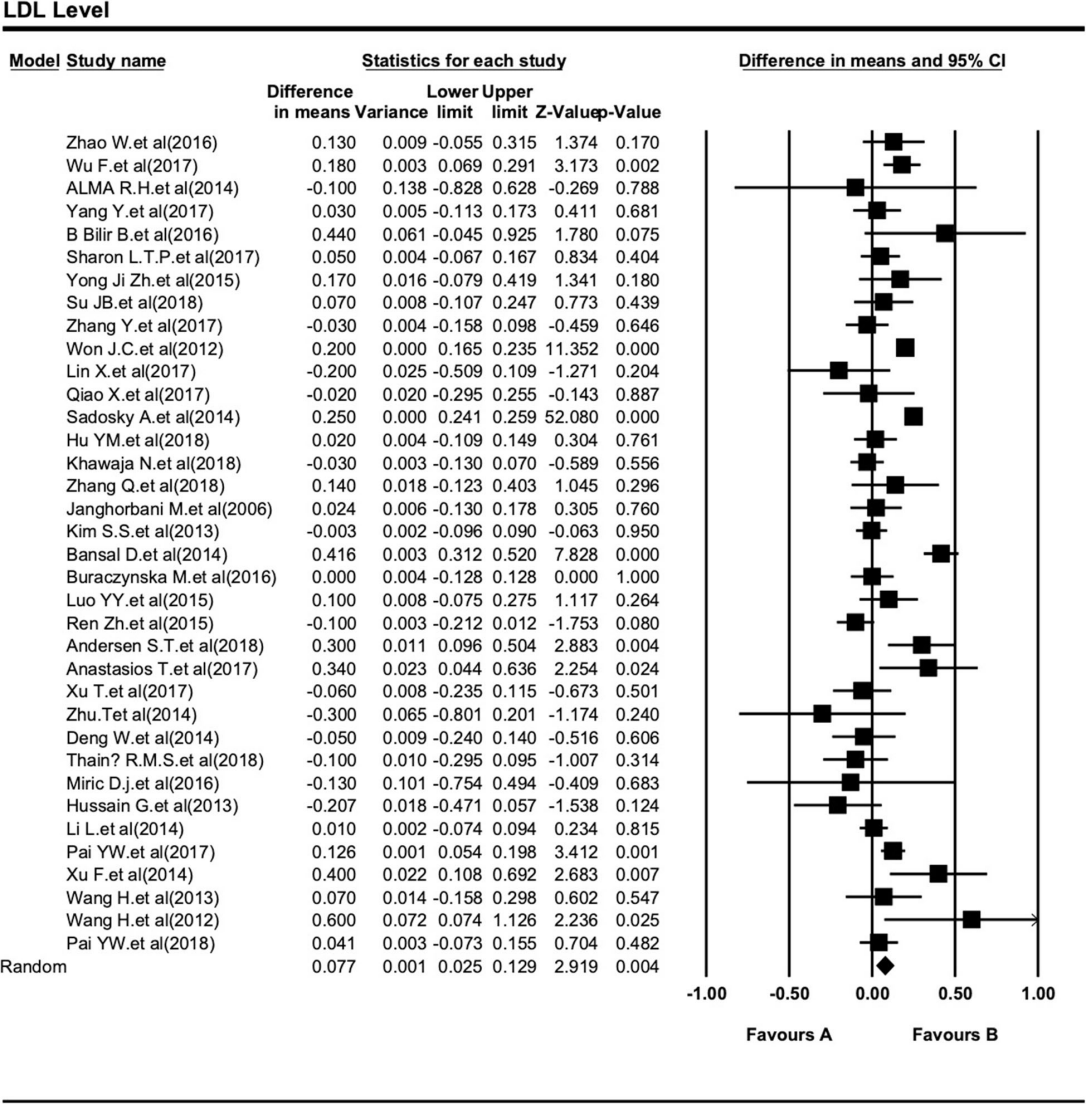
Forest plot of the association between serum LDL level and type 2 diabetic peripheral neuropathy. The differences between DPN and non-DPN groups calculated with standardized mean difference (SMD) with 95% confidence intervals (CIs) in the random effect model

We collected the mean and SD of LDL in both DPN and non-DPN patients of T2DM (Table 3). The Meta-analysis report on LDL (DPN = 2.78 ± 0.98 and non-DPN = 2.86 ± 0.91 mmol/L) showed that there was no significant difference between two groups’ age-matched participants at a high effect level (SMD = 0.08, 95% CI: 0.03–0.130.05; *P <* 0.001). In order to assess the influence of LDL level in the development DPN in T2DM patients, we collected SMD with 95% CIs of LDL level of 36 included studies, after analyzed all the studies; there was obvious heterogeneity in those 36 studies (I^2^ = 88.10%, Cochran Q-test *P <* 0.001) (Fig. 2).

**Table 3:** Association between LDL levels with the type 2 diabetic peripheral neuropathy risks

Subgroup analyses		N (%)	T2DM with DPN*	T2DM without DPN	SMD (95% CI) *	Heterogeneity	
			Mean + SD	Mean + SD		*P*-value**	I-squared
Ethnicity	America	3 (8.3)	2.54 + 1.02	2.61 + 0.85	0.07 (−0.24–0.37)	<0.001	84.92
	Asia	29 (80.6)	2.75 + 0.96	2.82 + 0.88	0.07 (0.01–0.12)	<0.001	78.97
	Europe	4 (11.2)	3.21 + 0.90	3.37 + 0.95	0.16 (− 0.06–0.38)	0.03	66.96
Study type	Case control	11 (30.6)	2.87 + 1.11	2.90 + 1.02	0.03 (− 0.05–0.11)	0.12	34.80
	Cohort	3 (8.3)	2.82 + 0.60	2.98 + 0.62	0.16 (− 0.05–0.37)	<0.001	84.15
	Cross-sectional	22 (61.1)	2.71 + 0.89	2.78 + 0.83	0.07 (0.01–0.13)	<0.001	80.52
Total (Random Effect Model)		36 (100)	2.78 + 0.98	2.86 + 0.91	0.08 (0.03–0.13)	<0.001	88.10

*The negative combined effect suggests that the LDL mean was higher in the non- type 2 diabetic peripheral neuropathy comparable group i.e. diabetes whereas the positive value would suggest greater LDL mean values in the type 2 diabetic peripheral neuropathy group

**P-values is calculated LDL mean between the T2DM with and without DPN

All LDL is reported in mmol/L

Abbreviations: N, number; T2DM, type-2 diabetes mellitus; DPN, diabetic peripheral neuropathy; SMD, standardized mean difference; Cl: Confidence interval

**Fig. 3:**
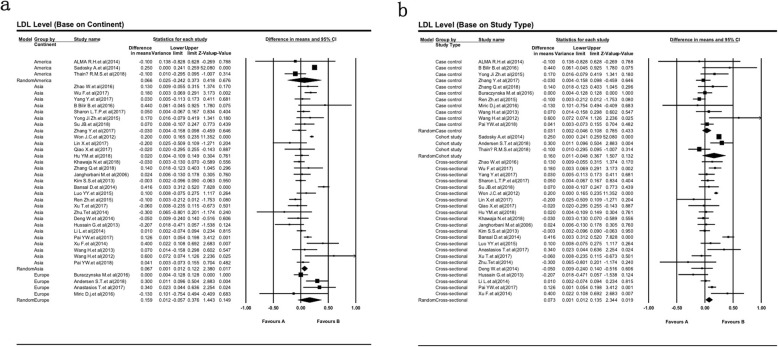
Summary receiver operating characteristic curve for serum LDL level and subgroup analysis based on race (**a**) and study design (**b**). Weights are from random effects analysis

The subgroup’s analysis conducted regarding the type of race and study design (Table 2). Figure 3a reveals none of the above covariates contributed to the heterogeneity (all *P >* 0.05). As shown in Table 2, the European DPN patients (SMD = 0.16, 95% CI: − 0.06 - 0.38) have higher serum level of LDL in compare with the American and Asian DPN patients (SMD = 0.07, 95% CI: − 0.24 - 0.37 and SMD = 0.07, 95% CI: 0.01–0.12; respectively). Furthermore, subgroup analysis of different study design showed more accuracy of cohort study design for the evaluation of serum LDL in type 2 diabetic peripheral neuropathy (Fig. 3b).

### Association of SBP level with DPN

**Fig. 4:**
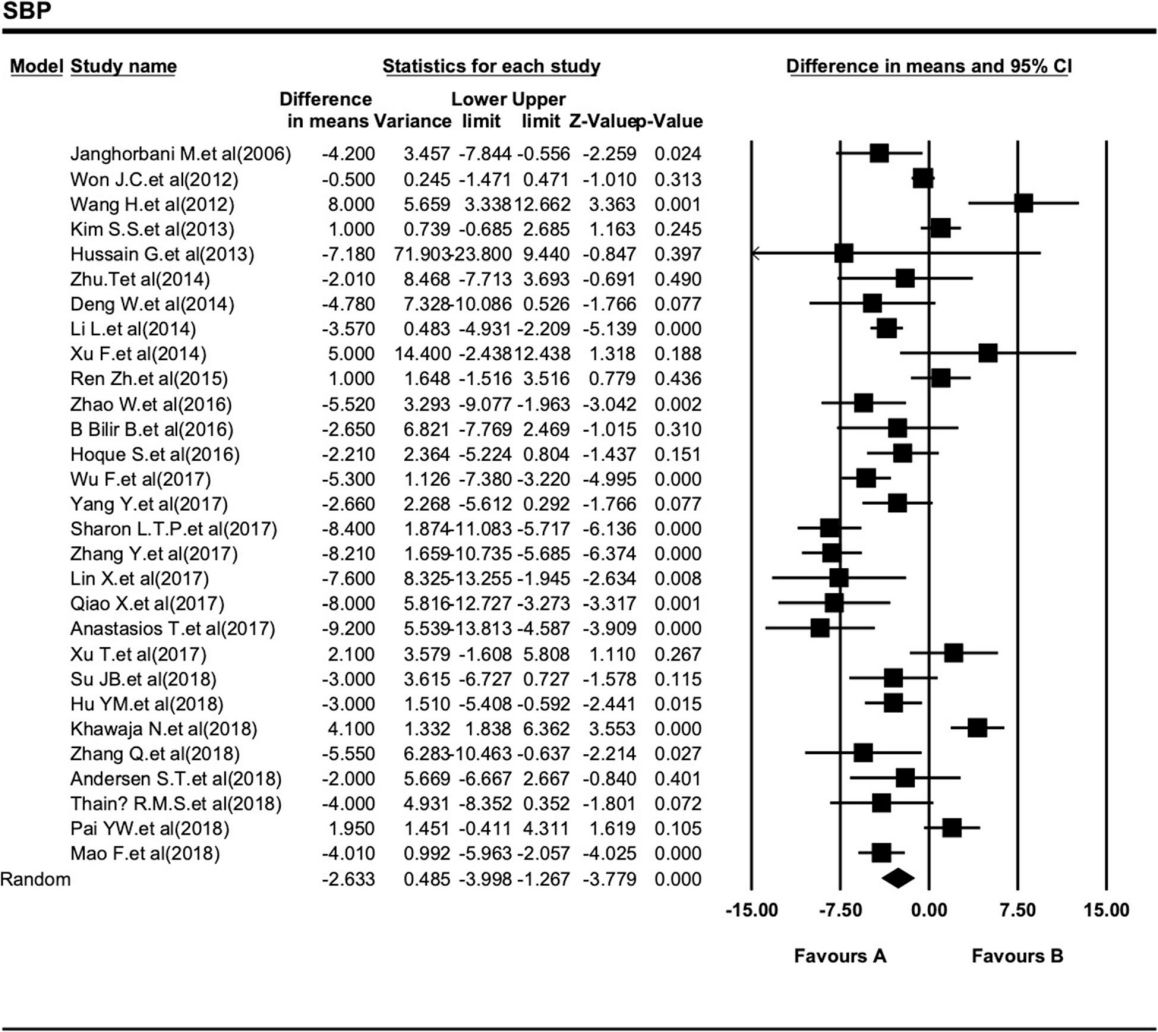
Forest plot of the association between SBP level and type 2 diabetic peripheral neuropathy. These plots show the prognostics accuracy of SBP for all objective response analysis. The differences between DPN and non-DPN groups calculated with standardized mean difference (SMD) with 95% confidence intervals (CIs) in the random effect model

We tried to evaluate the difference SBP level between DPN and non-DPN in patients with T2DM in Table 4. Also, the association between SBP and DPN level in patients with T2DM are shown in Fig. 4. This combined analysis of 29 studies indicates SBP level range with a higher heterogeneity I^2^ = 84.88% (Fig. 4). In comparison with the T2DM patients with DPN, the participants without DPN had significantly lower SBP levels (138.45 ± 18.50 mmHg vs 141.08 ± 19.10 mmHg). SBP level was associated with a statistically significant 2.6 fold reduce in non-DPN patients of T2DM when compared to the DPN group (SMD = − 2.63, 95% CI: − 4.00 - -1.27, *P <* 0.001; Table 4).

**Table 4:** Associations between SBP levels with the type 2 diabetic peripheral neuropathy risk

Subgroup analyses		N (%)	T2DM with DPN*	T2DM without DPN	SMD (95% CI)	Heterogeneity	
			Mean + SD	Mean + SD		*P*-value**	I-squared
Ethnicity	Asia	26	134.64 + 18.81	132.26 + 16.55	−2.38 (−3.81 - -0.95)	<0.001	85.70
	Europe	2	150.27 + 11.45	144.66 + 9.85	−5.61 (−12.66–1.45)	0.03	78.38
	America	1	146.00 + 23.00	142.00 + 22.00	−4.00 (−8.35–0.35)	NA	NA
Study type	Case control	6	134.52 + 18.59	133.58 + 17.99	−0.94 (− 5.49–3.61)	<0.001	91.33
	Cohort	2	148.07 + 21.20	145.00 + 20.10	−3.07 (−6.25–0.11)	0.54	0.00
	Cross-sectional	21	135.57 + 18.67	132.51 + 16.43	−3.06 (− 4.55 - -1.57)	<0.001	83.99
Total (Random Effect Model)		29 (100)	141.08 + 19.10	138.45 + 18.50	−2.63 (− 4.00 - -1.27)	<0.001	84.88

*The negative combined effect suggests that the LDL mean was higher in the non- type 2 diabetic peripheral neuropathy comparable group i.e. diabetes whereas the positive value would suggest greater LDL mean values in the type 2 diabetic peripheral neuropathy group

**P-values is calculated LDL mean between the T2DM with and without DPN

All LDL is reported in mmol/L

Abbreviations: N, number; T2DM, type-2 diabetes mellitus; DPN, diabetic peripheral neuropathy; SMD, standardized mean difference; Cl: Confidence interval

**Fig. 5:**
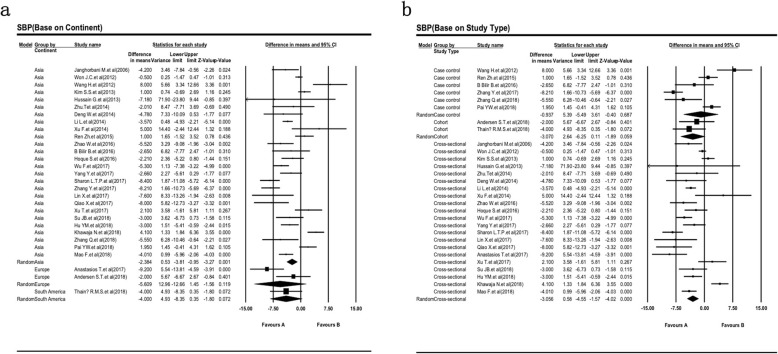
Sub group analysis to evaluation the difference of SBP level between DPN and non-DPN in patients with T2DM based of different race (**a**) and study design (**b**). Weights are from random effects analysis

Heterogeneity was noticeably decreased after the analysis of study design and race subgroup. There is low heterogeneity between studies which are cross-sectional study design (SMD = − 3.06, 95% CI: − 4.55 - -1.57, I^2^ = 83.99%; Fig. 5a) and performed in the Europe population (SMD = − 5.61, 95% CI: − 12.66 - 1.45, I^2^ = 78.38; Fig. 5b). Meanwhile, the highest significant SMD of SBP is more precise in the cross-sectional study design model. Thus, SBP may be a high-risk factor for the occurrence of DPN in European diabetic patients.

### Meta-regression results

**Fig. 6:**
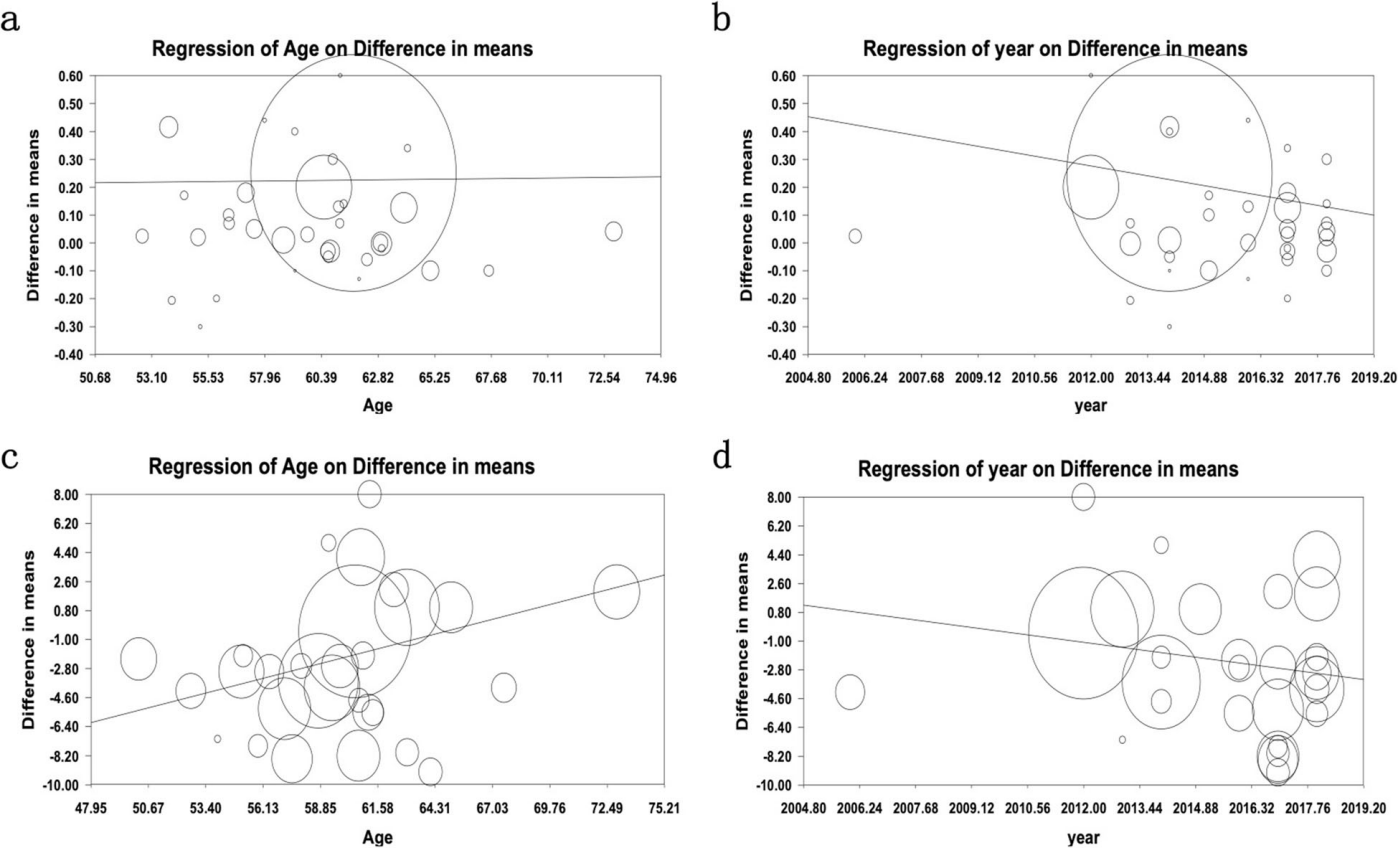
Meta regression results. Meta regression analysis for LDL based on age of participants (**a)** and year of publication (**b)** is in compare with meta-regression analysis for SBP based on age of participants (**c)** and year of publication (**d**). The size of each square is proportional to the percentage weight that each study contributed to the standardized mean difference (SDM) ratio. Weights are from random effects analysis

Meta-analysis regression was applied to investigate which factors determine heterogeneity among included individual studies in the meta-analysis. Meta-regression finding tried to clear the effects of the age of patients and the year of publication of articles on the average difference between the levels of LDL and SBP in both groups of study: T2DM patients with and without peripheral neuropathy (Fig. 6).

### The difference in collision risk of DPN drivers over time

By performing a meta-regression using the publications year, we tried to monitored change in collision risk of DPN drivers over time (Fig. 6a and b). The collision risk tends to decrease in the last 15 years (2004–2019) compared with the initial studies dealing specifically with this issue. In details, one-year increase in the average year of study, the difference of LDL level between the two groups were reduced 0.02 (B = − 0.02, 95% CI: − 0.03 - -0.01, *P <* 0.001; Fig. 6a) and difference in SBP levels between the two groups was reduced 0.32 (B = − 0.32, 95% CI: - 0.50 - - 0.14, *P <* 0.001; Fig. 6b). By running a meta-regression analysis, we found that there was a significantly decreased collision risk of DPN by drivers over time.

### Effect of age on collision risk in DPN

Effect of age on collision risk of DPN between both groups of T2DM with and without peripheral neuropathy is shown in Fig. 6c and d, respectively. Meta-regression results show that age had a significant influence on the collision risk in DPN drivers. By dividing the studies depending on their mean age into two groups, a one-unit increase in the average age of the patients, the difference in LDL level between the two groups increased 0.17 unit (Β = 0.001, 95% CI: − 0.004 - 0.006, *P =* 0.74; Fig. 6c); as well as a one-unit increase in the average age of the patient’s difference in SBP level between the two groups increased 33.0 unit. (Β = 0.33, 95%CI: 0.21–0.45, *P <* 0.001; Fig. 6d). In total, the elderly (47–75 years old) T2DM patients have a higher collision risk of DPN. Old age European T2DM patients have significantly high risk in the last 30 years for diabetes drivers that indicates LDL and SBP levels could be associated with increased risk of peripheral neuropathy in T2DM patients.

### Publication bias and sensitive analysis

**Fig. 7:**
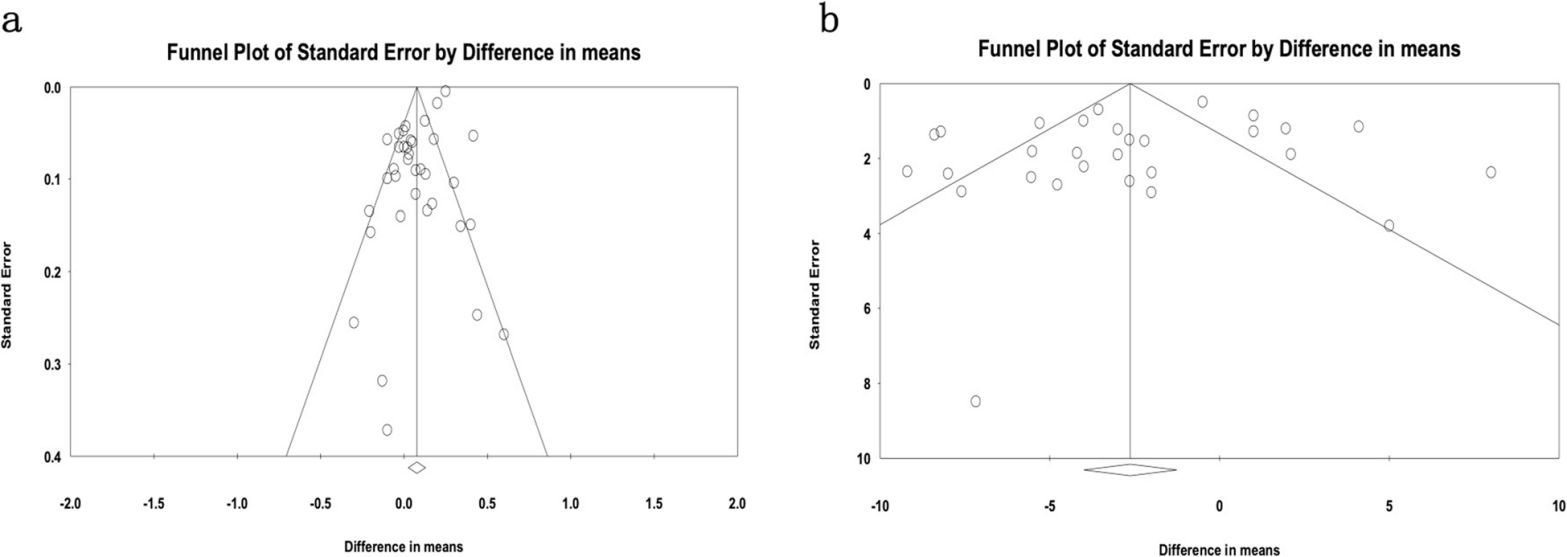
Contour-enhanced funnel plots for the detection of a publication bias of the LDL (**a**) and SBP (**b**). All enrolled 16 studies represent by each point for the specified association, individually. The size of each circle is proportional to the percentage weight that each study contributed to the standardized mean difference (SDM). These plots indicate that some studies were in significant areas where *P <* 0.01 (solid lines). Solid triangles refer to included studies and X’s refer to filled studies. The vertical axis represents standard error of logarithmic HR and the horizontal axis represents the SDM limits. CIs, confidence intervals; HR, hazard ratio

Investigations of publication bias and sensitivity were analyzed in the included literature with Begg’s and Egger’s regression test. The analysis was carried out by precluding a single study at a time and significant differences between events and hypnotizes (difference of mean LDL and SBP levels) (Fig. 7) [56]. Then, the Trim and Fill test was performed to find the studies missing impact on our results. This test indicates that the addition of three studies does not have any significant effects on our main findings (*n* = 3, Adjusted Mean Difference = 0.09, 95% CI: 0.04–0.14). The shape of funnel plot and Egger’s test provided no statistical evidence for publication bias of the LDL (t = − 0.92, *P* = 0.148, 38 study; Fig. 7a) and SBP (t = 1.11, *P* = 0.27, 29 study; Fig. 7b). Hence, there is no obvious proof for significant publication bias in our meta-analysis and meta-regression, which implies our stable and credible finding.

## Discussion

To the best of our knowledge, this is the first meta-analysis and meta-regression study assessed to distinguish T2DM patients with and without DPN according to the LDL and SBP profile. Our comprehensive meta-regression analysis has been conducted to identify the effects of T2DM patients age in collision risk of DPN in over time. Overall, we weighted a comprehensive analysis of the data from 36 clinical studies representing 355,438 T2DM patients. Significantly reduced SBP and increased LDL levels in non-DPN patients of T2DM patients highlight the potential role in peripheral neuropathy. The current interesting results indicate that SBP levels may be a high-risk factor for the occurrence of European DPN patients; which many cohort study design investigations could guaranty these finding in type 2 diabetic peripheral neuropathy. Furthermore, we find European T2DM patients have higher serum level of LDL in compare with the Asian DPN patients. Thus, LDL may be a high-risk factor for the occurrence of DPN in diabetic patients. On the other hand, results of the meta-analyses showed that elderly persons 65+ and 75+ years were more vulnerable than their respective counterparts using the pooled estimate for DPN. Of the study characteristics evaluated for age on collision risk, only the 47–75 years old T2DM patients have a significant risk for explaining prognostics accuracy of LDL and SBP.

It’s already well-established that T2DM patients that suffering from peripheral neuropathy have different LDL and SBP profiles that potentially influence the occurrence of DPN [22, 53–55]. Hypoglycemia is considered one of the main factors associated with an increased collision risk in DPN drivers. Similarly, some other physiology variables such as total cholesterol, albuminuria triglyceride, high-density lipoprotein cholesterol, hypertension, identified as predictors of DPN in other populations [11, 50, 57]. Reduced LDL level has been associated with diabetic nephropathy, neuropathy, and diabetic foot, and it has also been established to be an independent predictor of lower-extremity amputation in patients with type 2 diabetic foot wound. Several published meta-analyses have concerned to evaluate the dissimilarity of LDL and hypoglycemia to the prognosis risk for diabetes [48, 49]. Despite these competent studies, the glucose fluctuations in the interconnecting angiogenesis of the T2DM patients with neurotoxicity are not well-defined yet.

In compared with T2DM patients without neuropathy, oxidative stress, endothelial dysfunction, and the abnormal production of cytokines could be a strong possible reason for low LDL and SBP levels; which involved in the pathogenesis of painful diabetic neuropathy [41–43]. Patients with painful neuropathy had greater glycemic excursions, spatial abilities, myocardial damaging, psychomotor inactively, and cognitive abilities that have been linked to poorer diabetes control and other episodes’ disease like cardiac ischemia [52].

Our pooled results provide compelling evidence of a significant positive association between LDL and SBP and race. Recently, many studies showed diabetes patients from Europe have a lower collision risk compared with their Asian and American counterparts [50, 51, 58, 59]. Consequently, collision risk for DPN drivers is affected significantly by the race in which the studies have been performed. Definitely, future geographical cohort study need to as proving such a complement evidence association, and taking into account the race of this association could change the medical criteria of NPD [44–47].

A prospective observational study was carried out in the United Kingdom involving a large number of multiethnic T2DM to estimate the influence of SBP on microvascular complications. Results showed that there was a significant relationship of SBP with microvascular complications and observed that each 10 mmHg reduction in SBP decreased these complications by 11% (47). In other study comparing type 2 diabetics with and without DPN, it was found that SBP was higher in T2DM with DPN (48).

The large sample size, novelty, and standardized data compilation procedures are the main strengths of this study, which share the advantages of being specific and inexpensive of our finding. We should point out that there are a number of limitations in this investigation. We only included the papers in the English language, while published papers in other languages, especially Chinese and Russian, were ignored and absolutely causes selection bias. Inevitably, the majority of published studies are cross-sectional design study that did not disclose the information on patient preoperative or postoperative treatments and did not permit inferences regarding the causal relationship between clinical variables and DPN. Importantly, the different measurement techniques and cut-off uniform definition of LDL and SBP performed to be different for each study, which might also affect the precision of the estimate and raises some doubts about standardization. Fundamentally, the meta-analysis results were based on unadjusted estimates, because some studies did not provide detailed information of participants for each study to calculate the adjusted estimates, such as BMI, and physical inactivity, type of adjuvant therapy and generalizability. Certainly, the results of a current meta-analysis should be interpreted cautious and well-designed further longitudinal studies in large-scale, matched case-controls and functional studies are of great value to warrant these findings.

## Conclusion

Despite some limitations, the data of the present meta-analysis shows that high levels of SBP and LDL are two adaptable risk factors for DPN in European adults with T2DM. However, it has been determined that discovering age > 75 years in T2DM patients have a higher collision risk of DPN. Therefore, the LDL and SBP status could be associated with increased risk of peripheral neuropathy in T2DM patients.

## Supplementary information

**Additional file 1: Table S1.** Quality assessment of all relevant studies.

**Additional file 2: Figure S1.** Risk of bias graph. The overall risk of bias was regarded as low in all qualified studies, in terms of the QUADAS-2 assessment. The reviewers’ decisions about each risk of bias (**a**) and applicability concerns graph (**b**) presented as percentages across selected studies.

## Data Availability

The datasets used and/or analyzed during the current study are available from the corresponding author on reasonable request.

## Abbreviations

ADA: American diabetes association
AHRQ: Agency for healthcare research and quality
Cl: Confidence interval
DPN: Diabetic peripheral neuropathy
LDL: Low-density lipoprotein
mg/Dl: Milligram per deciliter
mmHg: Millimeter of mercury
mmol/L: Mill mole per liter
NR: Not reported
PRISMA: Preferred reporting items for systematic reviews and meta-analysis
QUADAS − 2: Quality assessment of diagnostic accuracy studies 2
SBP: Systolic blood pressure
SD: Standard deviation
SMD: Standardized mean difference
T2DM: Type-2 diabetes mellitus
