# Supplementary material for: Associations of serum low-density lipoprotein and systolic blood pressure levels with type 2 diabetic patients with and without peripheral neuropathy: systemic review, meta-analysis and meta-regression analysis of observational studies
Source: BMC Endocr Disord. 2019 Nov 25;19:125. doi: 10.1186/s12902-019-0453-5 (PMC6878654; doi:10.1186/s12902-019-0453-5)
Supplement: Supplementary file 1 — Additional file 1: Table S1. Quality assessment of all relevant studies. [file 12902_2019_453_MOESM1_ESM.docx]

**Additional file 1**

**Title:**

**Associations of serum low-density lipoprotein and systolic blood pressure levels with type 2 diabetic patients with and without peripheral neuropathy: Systemic review, meta-analysis and meta-regression analysis of observational studies**

**Syed Shah Zaman Haider Naqvi, Saber Imani, Hossein Hosseinifard, QingLian Wen, M. Naveed Shahzad, Iqra Ijaz, Youcai Deng, Man Guo and Yong Xu,**

**Inventory of Supplemental Information**

**- Supplementary Table 1‎ (Page 2-3)**

**- References (4-6)**

# Supplementary Table S1. Quality assessment of the included studies according to the Newcastle-Ottawa Scale (NOS)

| **Author**  **(Ref)** | **Year** | **Case** | | **Control** | | **Comparability** | | **Exposure** | | | | **NOS score** |
| --- | --- | --- | --- | --- | --- | --- | --- | --- | --- | --- | --- | --- |
|  |  | **Definition** | **Representativeness** | **Selection** | **Definition** | **Important factors** | **Other factors** | **Secure record** | **Blind** | **Method** | **Non-response rate** |  |
| Zhao W. et al (1) | 2016 | ★ | ★ | ★ | ★ | ★ | ★ | ☆ | ★ | ★ | ☆ | ‎8‎ |
| Wu F. et al (2) | 2017 | ★ | ★ | ★ | ★ | ★ | ★ | ★ | ☆ | ★ | ☆ | ‎8‎ |
| ALMA R.H. et al(3) | 2014 | ★ | ★ | ★ | ★ | ★ | ★ | ☆ | ★ | ★ | ☆ | ‎8‎ |
| Yang J. et al(4) | 2017 | ★ | ★ | ★ | ★ | ★ | ★ | ☆ | ☆ | ★ | ★ | ‎8‎ |
| B Bilir. Et al(5) | 2016 | ★ | ★ | ★ | ☆ | ★ | ★ | ★ | ☆ | ★ | ☆ | ‎7‎ |
| Sharon L.T.P. et al(6) | 2017 | ★ | ★ | ★ | ★ | ★ | ★ | ☆ | ★ | ★ | ★ | ‎8 |
| Yong Ji Zh. Et al (7) | 2015 | ★ | ★ | ★ | ★ | ★ | ★ | ☆ | ☆ | ★ | ★ | ‎8‎ |
| Su JB. et al(8) | 2018 | ★ | ★ | ★ | ☆ | ★ | ☆ | ★ | ★ | ★ | ★ | ‎8‎ |
| Zhang Y. et al(9) | 2017 | ★ | ★ | ★ | ☆ | ★ | ★ | ★ | ☆ | ★ | ☆ | ‎7‎ |
| Won J.C. et al (10) | 2012 | ★ | ★ | ★ | ★ | ★ | ★ | ★ | ☆ | ☆ | ☆ | ‎7‎ |
| Lin X. et al (11) | 2017 | ★ | ★ | ★ | ★ | ★ | ★ | ★ | ☆ | ★ | ☆ | ‎8‎ |
| Qiao X. et al (12) | 2017 | ★ | ★ | ★ | ★ | ★ | ☆ | ★ | ★ | ★ | ☆ | ‎8‎ |
| Sadosky A. et al (13) | 2014 | ★ | ☆ | ★ | ★ | ★ | ★ | ★ | ★ | ★ | ☆ | ‎8‎ |
| Hu YM. Et al (14) | 2018 | ★ | ★ | ★ | ☆ | ★ | ★ | ★ | ★ | ☆ | ☆ | ‎7‎ |
| Khawaja N. et al (15) | 2018 | ★ | ★ | ★ | ★ | ★ | ★ | ★ | ☆ | ★ | ☆ | 8 |
| Zhang Q. et al (16) | 2018 | ★ | ★ | ★ | ★ | ★ | ★ | ★ | ☆ | ★ | ☆ | 8 |
| Janghorbani M. et al (17) | 2006 | ★ | ★ | ★ | ★ | ★ | ★ | ★ | ☆ | ★ | ☆ | 8 |
| Kim S.S.et al (18) | 2013 | ★ | ★ | ★ | ★ | ★ | ★ | ☆ | ★ | ★ | ☆ | 8 |
| Bansal D. et al (19) | 2014 | ★ | ★ | ★ | ★ | ★ | ★ | ★ | ☆ | ★ | ☆ | 8 |
| Buraczynska M. et al (20) | 2016 | ★ | ★ | ★ | ★ | ★ | ★ | ☆ | ☆ | ★ | ☆ | 7 |
| Luo YY.et al(21) | 2015 | ★ | ★ | ★ | ★ | ★ | ★ | ★ | ★ | ★ | ★ | 8 |
| Ren Zh.et al (22) | 2015 | ★ | ★ | ★ | ★ | ★ | ★ | ★ | ☆ | ☆ | ☆ | 7 |
| Andersen S.T.et al (23) | 2018 | ★ | ★ | ★ | ★ | ★ | ★ | ★ | ★ | ★ | ★ | 8 |
| Anastasios T. et al (24) | 2017 | ★ | ★ | ★ | ★ | ★ | ★ | ★ | ★ | ☆ | ☆ | 8 |
| Xu T.et al (25) | 2017 | ★ | ★ | ★ | ★ | ★ | ★ | ★ | ☆ | ★ | ☆ | 8 |
| Zhu T. et al (26) | 2014 | ★ | ★ | ★ | ★ | ★ | ★ | ★ | ☆ | ★ | ☆ | 8 |
| Deng W.et al (27) | 2014 | ★ | ★ | ★ | ★ | ★ | ★ | ★ | ☆ | ★ | ☆ | 8 |
| Thainá R.M.S.et al (28) | 2018 | ★ | ★ | ★ | ★ | ★ | ★ | ☆ | ☆ | ★ | ☆ | 7 |
| Miric D.J. et al (29) | 2016 | ★ | ★ | ★ | ★ | ★ | ★ | ☆ | ★ | ☆ | ☆ | 7 |
| Hussain G. et al (30) | 2013 | ★ | ★ | ★ | ★ | ★ | ★ | ★ | ☆ | ☆ | ☆ | 7 |
| Li L. et al (31) | 2014 | ★ | ★ | ★ | ★ | ★ | ★ | ★ | ☆ | ★ | ☆ | 8 |
| Pai Y.W. et al (32) | 2018 | ★ | ★ | ★ | ★ | ★ | ★ | ★ | ☆ | ☆ | ☆ | 7 |
| Xu F (33) | 2014 | ★ | ★ | ★ | ★ | ★ | ★ | ★ | ★ | ☆ | ☆ | 8 |
| Wang H.et al (34) | 2013 | ★ | ★ | ★ | ★ | ★ | ★ | ☆ | ★ | ☆ | ☆ | 7 |
| Wang H.et al (35) | 2012 | ★ | ★ | ★ | ★ | ★ | ★ | ★ | ★ | ☆ | ☆ | 8 |
| Pai Y.W. et al (36) | 2018 | ★ | ★ | ★ | ★ | ★ | ★ | ★ | ★ | ☆ | ☆ | 8 |
| Mao F.et al (37) | 2018 | ★ | ☆ | ★ | ★ | ★ | ★ | ☆ | ★ | ★ | ☆ | ‎8‎ |
| Hoque S. et al (38) | 2016 | ★ | ★ | ★ | ★ | ★ | ★ | ☆ | ★ | ★ | ☆ | ‎8‎ |
| ★, score value=1; ☆, score value=0; The specific item information is available from http://www.ohri.ca/programs/clinical_epidemiology/oxford.asp. | | | | | | | | | | | | |

**References**

1. Zhao W, Zeng H, Zhang X, Liu F, Pan J, Zhao J, et al. A high thyroid stimulating hormone level is associated with diabetic peripheral neuropathy in type 2 diabetes patients. Diabetes Res Clin Pract. 2016;115:122-9.

2. Wu F, Jing Y, Tang X, Li D, Gong L, Zhao H, et al. Anemia: an independent risk factor of diabetic peripheral neuropathy in type 2 diabetic patients. Acta diabetologica. 2017;54(10):925-31.

3. Rosales-Hernandez A, Cheung A, Podgorny P, Chan C, Toth C. Absence of clinical relationship between oxidized low density lipoproteins and diabetic peripheral neuropathy: a case control study. Lipids in health and disease. 2014;13:32.

4. Yang J, Yan PJ, Wan Q, Li H. Association between Hemoglobin Levels and Diabetic Peripheral Neuropathy in Patients with Type 2 Diabetes: A Cross-Sectional Study Using Electronic Health Records. Journal of diabetes research. 2017;2017:2835981.

5. Bilir B, Ekiz Bilir B, Yilmaz I, Soysal Atile N, Yildirim T, Kara SP, et al. Association of apelin, endoglin and endocan with diabetic peripheral neuropathy in type 2 diabetic patients. European review for medical and pharmacological sciences. 2016;20(5):892-8.

6. Pek SLT, Sum CF, Yeoh LY, Lee SBM, Tang WE, Lim SC, et al. Association of apolipoprotein-CIII (apoC-III), endothelium-dependent vasodilation and peripheral neuropathy in a multi-ethnic population with type 2 diabetes. Metabolism: clinical and experimental. 2017;72:75-82.

7. Ji ZY, Li HF, Lei Y, Rao YW, Tan ZX, Liu HJ, et al. Association of adiponectin gene polymorphisms with an elevated risk of diabetic peripheral neuropathy in type 2 diabetes patients. Journal of diabetes and its complications. 2015;29(7):887-92.

8. Su JB, Zhao LH, Zhang XL, Cai HL, Huang HY, Xu F, et al. HbA1c variability and diabetic peripheral neuropathy in type 2 diabetic patients. Cardiovascular diabetology. 2018;17(1):47.

9. Zhang Y, Jiang Y, Shen X, Yan S. Can both normal and mildly abnormal albuminuria and glomerular filtration rate be a danger signal for diabetic peripheral neuropathy in type 2 diabetes mellitus? Neurological sciences : official journal of the Italian Neurological Society and of the Italian Society of Clinical Neurophysiology. 2017;38(8):1381-90.

10. Won JC, Kwon HS, Kim CH, Lee JH, Park TS, Ko KS, et al. Prevalence and clinical characteristics of diabetic peripheral neuropathy in hospital patients with Type 2 diabetes in Korea. Diabetic medicine : a journal of the British Diabetic Association. 2012;29(9):e290-6.

11. Lin X, Xu L, Zhao D, Luo Z, Pan S. Correlation between serum uric acid and diabetic peripheral neuropathy in T2DM patients. Journal of the neurological sciences. 2018;385:78-82.

12. Qiao X, Zheng H, Zhang S, Liu S, Xiong Q, Mao F, et al. C-peptide is independent associated with diabetic peripheral neuropathy: a community-based study. Diabetology & metabolic syndrome. 2017;9:12.

13. Sadosky A, Mardekian J, Parsons B, Hopps M, Bienen EJ, Markman J. Healthcare utilization and costs in diabetes relative to the clinical spectrum of painful diabetic peripheral neuropathy. Journal of diabetes and its complications. 2015;29(2):212-7.

14. Hu YM, Zhao LH, Zhang XL, Cai HL, Huang HY, Xu F, et al. Association of glycaemic variability evaluated by continuous glucose monitoring with diabetic peripheral neuropathy in type 2 diabetic patients. Endocrine. 2018;60(2):292-300.

15. Khawaja N, Abu-Shennar J, Saleh M, Dahbour SS, Khader YS, Ajlouni KM. The prevalence and risk factors of peripheral neuropathy among patients with type 2 diabetes mellitus; the case of Jordan. Diabetology & metabolic syndrome. 2018;10:8.

16. Zhang Q, Ji L, Zheng H, Li Q, Xiong Q, Sun W, et al. Low serum phosphate and magnesium levels are associated with peripheral neuropathy in patients with type 2 diabetes mellitus. Diabetes Res Clin Pract. 2018;146:1-7.

17. Janghorbani M, Rezvanian H, Kachooei A, Ghorbani A, Chitsaz A, Izadi F, et al. Peripheral neuropathy in type 2 diabetes mellitus in Isfahan, Iran: prevalence and risk factors. Acta neurologica Scandinavica. 2006;114(6):384-91.

18. Kim SS, Won JC, Kwon HS, Kim CH, Lee JH, Park TS, et al. Prevalence and clinical implications of painful diabetic peripheral neuropathy in type 2 diabetes: results from a nationwide hospital-based study of diabetic neuropathy in Korea. Diabetes Res Clin Pract. 2014;103(3):522-9.

19. Bansal D, Gudala K, Muthyala H, Esam HP, Nayakallu R, Bhansali A. Prevalence and risk factors of development of peripheral diabetic neuropathy in type 2 diabetes mellitus in a tertiary care setting. J Diabetes Investig. 2014;5(6):714-21.

20. Buraczynska M, Buraczynska K, Dragan M, Ksiazek A. Pro198Leu Polymorphism in the Glutathione Peroxidase 1 Gene Contributes to Diabetic Peripheral Neuropathy in Type 2 Diabetes Patients. Neuromolecular medicine. 2017;19(1):147-53.

21. Luo YY, Zhao J, Han XY, Zhou XH, Wu J, Ji LN. Relationship Between Serum Zinc Level and Microvascular Complications in Patients with Type 2 Diabetes. Chinese medical journal. 2015;128(24):3276-82.

22. Ren Z, Ji N, Jia K, Wang L, Gu HF, Ma J. Association of the intercellular adhesion molecule-1 gene polymorphisms with type 2 diabetes and diabetic peripheral neuropathy in a Chinese Han population. Genes & Genomics. 2015;37(1):69-75.

23. Andersen ST, Witte DR, Dalsgaard EM, Andersen H, Nawroth P, Fleming T, et al. Risk Factors for Incident Diabetic Polyneuropathy in a Cohort With Screen-Detected Type 2 Diabetes Followed for 13 Years: ADDITION-Denmark. Diabetes Care. 2018;41(5):1068-75.

24. Tentolouris A, Eleftheriadou I, Grigoropoulou P, Kokkinos A, Siasos G, Ntanasis-Stathopoulos I, et al. The association between pulse wave velocity and peripheral neuropathy in patients with type 2 diabetes mellitus. Journal of diabetes and its complications. 2017;31(11):1624-9.

25. Xu T, Weng Z, Pei C, Yu S, Chen Y, Guo W, et al. The relationship between neutrophil-to-lymphocyte ratio and diabetic peripheral neuropathy in Type 2 diabetes mellitus. Medicine. 2017;96(45):e8289.

26. Zhu T, Meng Q, Ji J, Lou X, Zhang L. Toll-like receptor 4 and tumor necrosis factor-alpha as diagnostic biomarkers for diabetic peripheral neuropathy. Neuroscience letters. 2015;585:28-32.

27. Deng W, Dong X, Zhang Y, Jiang Y, Lu D, Wu Q, et al. Transcutaneous oxygen pressure (TcPO(2)): a novel diagnostic tool for peripheral neuropathy in type 2 diabetes patients. Diabetes Res Clin Pract. 2014;105(3):336-43.

28. Santos TRM, Melo JV, Leite NC, Salles GF, Cardoso CRL. Usefulness of the vibration perception thresholds measurement as a diagnostic method for diabetic peripheral neuropathy: Results from the Rio de Janeiro type 2 diabetes cohort study. Journal of diabetes and its complications. 2018;32(8):770-6.

29. Miric DJ, Kisic BM, Filipovic-Danic S, Grbic R, Dragojevic I, Miric MB, et al. Xanthine Oxidase Activity in Type 2 Diabetes Mellitus Patients with and without Diabetic Peripheral Neuropathy. Journal of diabetes research. 2016;2016:4370490.

30. Hussain G, Rizvi SA, Singhal S, Zubair M, Ahmad J. Cross sectional study to evaluate the effect of duration of type 2 diabetes mellitus on the nerve conduction velocity in diabetic peripheral neuropathy. Diabetes & metabolic syndrome. 2014;8(1):48-52.

31. Li L, Chen J, Wang J, Cai D. Prevalence and risk factors of diabetic peripheral neuropathy in Type 2 diabetes mellitus patients with overweight/obese in Guangdong province, China. Primary care diabetes. 2015;9(3):191-5.

32. Pai YW, Lin CH, Lee IT, Chang MH. Prevalence and biochemical risk factors of diabetic peripheral neuropathy with or without neuropathic pain in Taiwanese adults with type 2 diabetes mellitus. Diabetes & metabolic syndrome. 2018;12(2):111-6.

33. Xu F, Zhao LH, Su JB, Chen T, Wang XQ, Chen JF, et al. The relationship between glycemic variability and diabetic peripheral neuropathy in type 2 diabetes with well-controlled HbA1c. Diabetology & metabolic syndrome. 2014;6(1):139.

34. Wang H, Fan D, Zhang Y. Angiogenin gene polymorphism: A risk factor for diabetic peripheral neuropathy in the northern Chinese Han population. Neural regeneration research. 2013;8(36):3434-40.

35. Wang H, Fan D, Hong T. Is the C677T polymorphism in methylenetetrahydrofolate reductase gene or plasma homocysteine a risk factor for diabetic peripheral neuropathy in Chinese individuals? Neural regeneration research. 2012;7(30):2384-91.

36. Pai YW, Lin CH, Lee IT, Chang MH. Variability of fasting plasma glucose and the risk of painful diabetic peripheral neuropathy in patients with type 2 diabetes. Diabetes & metabolism. 2018;44(2):129-34.

37. Fei Mao XZ, Siying Liu, Xiaona Qiao, Hangping Zheng, Bin Lu, Yiming Li. Age as an Independent Risk Factor for Diabetic Peripheral Neuropathy in Chinese Patients with Type 2 Diabetes. Aging and disease.0-.

38. Hoque S, A Muttalib M, Islam M, Happy T. Evaluation of Different HbA1c Levels to Assess the Risk of Peripheral Neuropathy Among Type 2 Diabetic Patients Along With Other Conventional Risk Factors2017. 95 p.
