# Supplementary material for: Associations of serum low-density lipoprotein and systolic blood pressure levels with type 2 diabetic patients with and without peripheral neuropathy: systemic review, meta-analysis and meta-regression analysis of observational studies
Source: BMC Endocr Disord. 2019 Nov 25;19:125. doi: 10.1186/s12902-019-0453-5 (PMC6878654; doi:10.1186/s12902-019-0453-5)
Supplement: Supplementary file 2 — Additional file 2: Figure S1. Risk of bias graph. The overall risk of bias was regarded as low in all qualified studies, in terms of the QUADAS-2 assessment. The reviewers’ decisions about each risk of bias (a) and applicability concerns graph (b) presented as percentages across selected studies. [file 12902_2019_453_MOESM2_ESM.docx]

**Additional file 2**

**Title:**

**Associations of serum low-density lipoprotein and systolic blood pressure levels with type 2 diabetic patients with and without peripheral neuropathy: Systemic review, meta-analysis and meta-regression analysis of observational studies**

**Syed Shah Zaman Haider Naqvi, Saber Imani, Hossein Hosseinifard, QingLian Wen, M. Naveed Shahzad, Iqra Ijaz, Youcai Deng, Man Guo and Yong Xu,**

**Inventory of Supplemental Information**

**- Supplementary Fig. 1‎ (Page 2)**

**Supplementary Fig. S1.** Risk of bias graph. The overall risk of bias was regarded as low in all qualified studies, in terms of the QUADAS-2 assessment. The reviewers' decisions about each risk of bias (**a**) and applicability concerns graph (**b**) presented as percentages across selected studies.

**
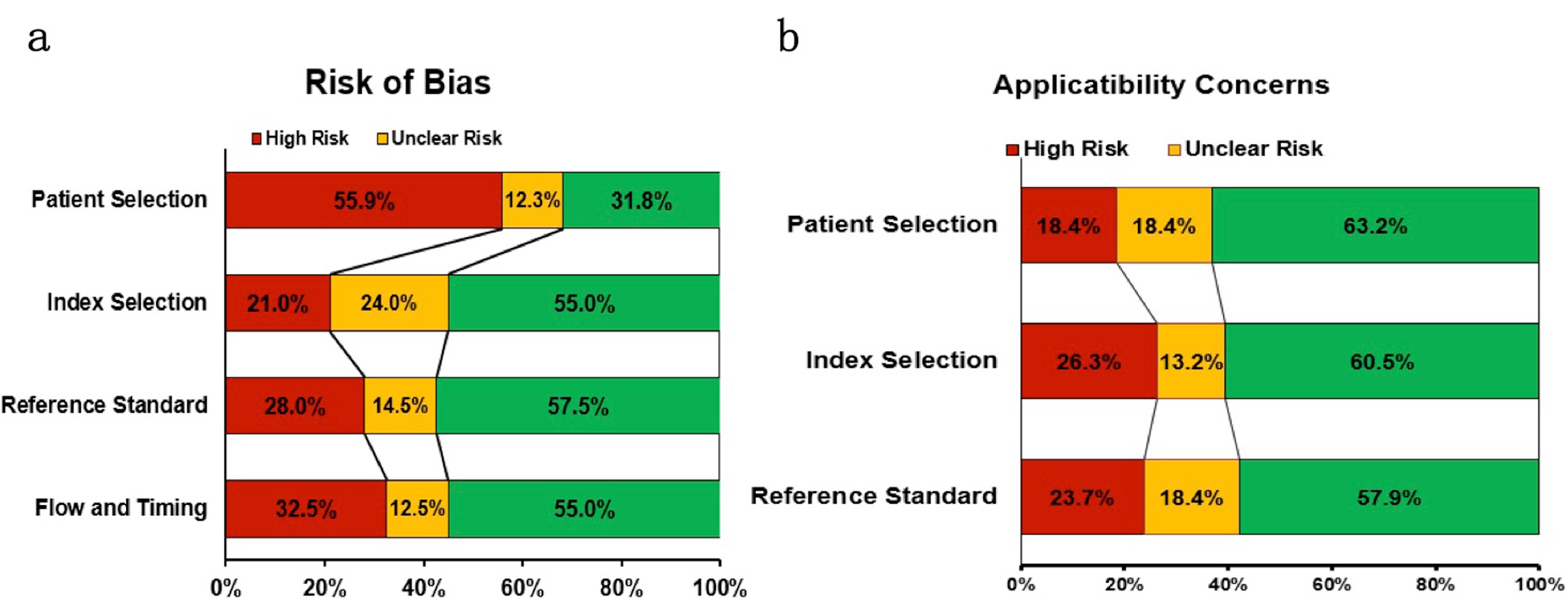
**
